# Supplementary material for: Scalable Causal Structure Learning: Scoping Review of Traditional and Deep Learning Algorithms and New Opportunities in Biomedicine
Source: JMIR Med Inform. 2023 Jan 17;11:e38266. doi: 10.2196/38266 (PMC9890349; doi:10.2196/38266)
Supplement: Multimedia Appendix 1 [file medinform_v11i1e38266_app1.docx]

**Multimedia Appendix 1**

| PC (named after Peter Spirtes and Clark Glymour) | R-pcalg, R-bnlean, Tetrad, Python-pgmpy |
| --- | --- |
| IC (Inductive Causation) | Python-causality |
| FCI (Fast Causal Inference) | R-pcalg, Tetrad |
| GES (Greedy Equivalence Search) | R-pcalg, Tetrad |
| Fast GES | Tetrad |
| K2 | [github.com/ruteee/K2-Algorithm](http://github.com/ruteee/K2-Algorithm) |
| Max-Min Hill Climbing (MMHC) | R-mmhc, Python-pgmpy, R-bnlean |
| LiNGAM (Linear Non-Gaussian Acyclic Model) | R-pcalg, Tetrad |
| GRAN-DAG | [github.com/kurowasan/GraN-DAG](https://github.com/kurowasan/GraN-DAG) |
| DAG-GNN | [github.com/fishmoon1234/DAG-GNN](https://github.com/fishmoon1234/DAG-GNN) |
| NOTEARS | [github.com/xunzheng/notears](https://github.com/xunzheng/notears)  [github.com/skypea/DAG_No_Fear](https://github.com/skypea/DAG_No_Fear) |
| GAE | [github.com/huawei-noah/trustworthyAI/tree/master/Causal_Structure_Learning/GAE_Causal_Structure_Learning](https://github.com/huawei-noah/trustworthyAI/tree/master/Causal_Structure_Learning/GAE_Causal_Structure_Learning) |
| RL-BIC | [github.com/huawei-noah/trustworthyAI/tree/master/Causal_Structure_Learning/Causal_Discovery_RL](https://github.com/huawei-noah/trustworthyAI/tree/master/Causal_Structure_Learning/Causal_Discovery_RL) |
| SAM | [github.com/Diviyan-Kalainathan/SAM](http://github.com/Diviyan-Kalainathan/SAM) |
| CGNN | [github.com/GoudetOlivier/CGNN](https://github.com/GoudetOlivier/CGNN) |

Table S1: List of tools for causal structure discovery.
